# Regulation of MAP Kinase signaling by the insulin-like growth factor pathway during *C. elegans* vulval development

**DOI:** 10.17912/micropub.biology.001557

**Published:** 2025-03-21

**Authors:** Matthew Eroglu, W. Brent Derry

**Affiliations:** 1 Department of Molecular Genetics, University of Toronto, Toronto, Ontario, Canada; 2 Developmental and Stem Cell Biology Program, Hospital for Sick Children, Toronto, Ontario, Canada

## Abstract

Organ development depends on multiple signaling pathways working in concert to specify cell fates. Improper activity or inactivity of specific signaling pathways such as EGF-Ras-MAPK can lead to dedifferentiation and cancer. In *C. elegans* , gain of function mutations in Ras/ *let-60* lead to ectopic development of multiple ventral vulva-like lesions resembling tumors. However, this phenotype depends on normal insulin-like growth factor (IGF) signaling. Here, we probe how factors downstream of the IGF receptor *daf-2* modify Ras signaling. These investigations led us to identify regulators of cell fate such as the Zinc finger protein encoding gene *mstr-1* ( *F22D6.2* ), homologous to mammalian *Zfand3* / *5* / *6* .

**Figure 1. Multiple regulators of activated Ras/MAPK signaling downstream of the IGF receptor in C. elegans:**
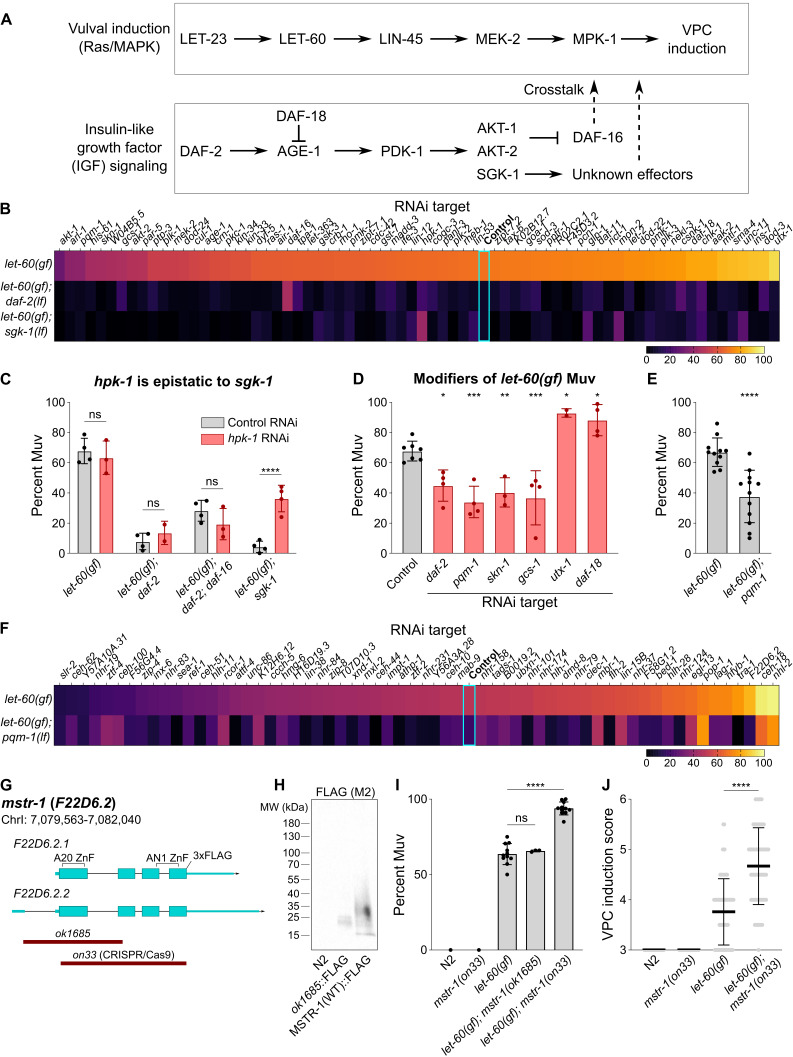
**Figure 1. (A)** Overview of Ras signaling in *C. elegans* which induces vulval differentiation (top) and insulin-like growth factor (IGF) signaling (bottom). **(B)** Penetrance of Multivulva (Muv) after RNAi treatment in the indicated genetic backgrounds. *let-60 (gf)* refers to the *n1046* allele; *daf-2 (lf)* refers to the *e1370* allele; *sgk-1 (lf)* refers to the *ok538* allele. Scale, percent Muv. **(C)** Worms of indicated genotypes were treated with *hpk-1* or control RNAi. ns, not significant; **** *P <* 0.0001; two-way ANOVA with Sidak's correction. Points, biological replicates; error bars, S.D. **(D)** Penetrance of Muv was quantified in *let-60 ( n1046 )***** worms treated with the indicated RNAi. * *P* < 0.05, ** *P* < 0.01, *** *P* < 0.001; one way ANOVA with Dunnett's correction. Points, biological replicates; error bars, S.D. **(E)** Muv penetrance in indicated genetic backgrounds. **** *P <* 0.0001, two-tailed t-test. Points, biologically unique populations; error bars, S.D. **(F)** Penetrance of Muv after RNAi treatment in the indicated genetic backgrounds. *let-60 (gf)* refers to the *n1046* allele; *pqm-1 (lf)* refers to the *ok485* allele. Scale, percent Muv. **(G)** Overview of the *mstr-1* genomic locus and the two deletions alleles used in **H** and **I** . **(H)** Western blot of WT and *ok1685* mutant MSTR-1 ::3xFLAG probed with anti-FLAG antibody. **(I)** Penetrance of Muv in indicated genetic backgrounds. ns, not significant; **** *P <* 0.0001; one-way ANOVA with Dunnett's correction excluding N2 and *mstr-1 ( on33 )* single mutants. Points, biologically unique populations; error bars, S.D. **(J)** Scoring of vulval induction in indicated genetic backgrounds. **** *P <* 0.0001, two-tailed t-test excluding N2 and *mstr-1 ( on33 )* single mutants. Points, individual worms; error bars, S.D.

## Description

The *Caenorhabditis elegans* vulva is one of the most comprehensively studied organs, work on which has revealed how multiple signaling networks including Wnt/β-catenin, Notch, and EGF/Ras/MAPK cooperate to specify then sculpt a structure that enables egg-laying in adult hermaphrodite worms. The adult vulva is an organ derived from the epidermis that is comprised of 22 cells that assemble into seven stacked rings forming a bridge between the worm's uterus and the external environment (Schindler and Sherwood 2013). These develop from 3 of 6 equipotent vulval precursor cells (VPCs) that are induced by an epidermal growth factor (EGF/ LIN-3 ) signal from the anchor cell which activates its receptor (EGFR/ LET-23 ) in the closest VPCs, inducing them to undergo 3 rounds of cell division to form the vulva (Hill and Sternberg 1992). In contrast, uninduced VPCs only divide once before fusing with the epidermis, except for P3.p which fuses before dividing in 50% of worms. In the induced VPCs, EGFR/ LET-23 activates the Ras homolog LET-60 , which then activates the MAP Kinase ERK/ MPK-1 through Raf/ LIN-45 and MEK/ MEK-2 ( **Fig. 1A** ). MPK-1 phosphorylates the ETS transcription factor ELK1/ LIN-1 , inactivating it and inducing vulval differentiation. Ablation of any MAPK component prevents vulval development, whereas gain of function (gf) mutations in the pathway lead to ectopic induction of VPCs, leading to more than 3 VPCs adopting vulval fates and development of additional vulva-like structures, a phenotype termed Multivulva (Muv).

Notably, mutations activating the MAPK pathway are partially penetrant, with *let-60 ( n1046 gf)* worms displaying ~70% penetrance, the *let-60 (ga89gf)* temperature sensitive allele displaying ~55% penetrance at restrictive temperatures (Ferguson and Horvitz 1985; Eisenmann and Kim 1997; Yoder et al. 2004), and *lin-45 gf* overexpressing worms displaying ~77% penetrance of Muv (Yoder et al. 2004). In contrast, loss of function of the MAPK target *lin-1* leads to a 100% penetrance of Muv (Beitel et al. 1995). Various gene ablations can enhance or suppress the penetrance of Muv in *let-60 (gf)* worms without affecting wild type vulval development. Collectively, these suggest that from Ras to MPK-1 , a signaling threshold must be met for a VPC to adopt an induced fate. This is similar to cancer-causing Ras *gf* mutations in mammalian models, where not every mutant cell adopts a cancerous fate and not every carrier individual develops cancer (Muñoz-Maldonado, Zimmer, and Medová 2019). Understanding how thresholds regulating MAPK output are established may enable better prediction of cell fates as well as elucidate how organs normally develop robustly in wild-type organisms, with relatively little variation in overall form.

The Muv phenotype of Ras/ *let-60* (gf) mutants depends on normal insulin-like growth factor (IGF) signaling ( **Fig. 1A** ). Loss of the IGF receptor *daf-2* or the downstream kinases *akt-1 and 2* or *sgk-1* leads to near-complete suppression of the Ras/ *let-60* Muv phenotype, but does not affect normal vulval development in wild type worms (Battu, Hoier, and Hajnal 2003; Hall 2014). This suppression at least partially depends on the downstream FOXO transcription factor DAF-16 (Hall 2014; Subramanian et al. 2021). However, *let-60 (gf); daf-2 ; daf-16* triple mutants are not fully rescued to *let-60 (gf)* single mutant levels, indicating that a *daf-16* independent effector of IGF signaling may be responsible for the remaining suppression of *let-60 (gf)* by loss of *daf-2* . Furthermore, preliminary evidence indicates that this *daf-16* independent arm may be downstream of *sgk-1* specifically, as *daf-16* RNAi restores Muv in *let-60 (gf); daf-2* worms but not in *let-60 ; sgk-1* worms ( **Fig. 1B** ). Likewise, loss of *daf-16* rescues the resistance of *daf-2* worms to triggering DNA damage induced germ cell apoptosis but does not affect a similar *sgk-1* phenotype (Perrin et al. 2013).

Here, we sought to identify putative FOXO/ *daf-16* independent regulators of MAPK signaling downstream of the IGFR/ *daf-2* pathway. We began our analysis by RNAi knockdown of genes that were experimentally shown or computationally predicted to interact genetically or physically with *sgk-1* as annotated on WormBase (Sternberg et al. 2024). These knockdowns were carried out in several mutant backgrounds ( **Fig. 1B** ): (1) *let-60 ( n1046 gf)* worms to look for enhancement or suppression of the Muv phenotype; (2) *let-60 ( n1046 gf); daf-2 ( e1370 lf)* worms to look for rescue of suppression by *daf-2* loss *;* and (3) *let-60 ; daf-2 ; sgk-1 (e538lf)* to look for rescue of suppression by *sgk-1* loss *.* Only genes present in the Ahringer lab RNAi library were screened (Kamath et al. 2003). Consistent with prior reports, among the modifiers we observed suppression of *let-60 (gf)* Muv by knockdown of *akt-1 , akt-2* and *age-1* as well as enhancement of Muv by *daf-18* (Hall 2014; Nakdimon et al. 2012). Likewise, while *daf-16* RNAi restored Muv in *let-60 (gf); daf-2 (lf)* worms, it had no influence on *let-60 (gf); sgk-1 (lf)* worms *.* Notably, RNAi knockdown of the homeodomain interacting protein kinase HIPK/ *hpk-1* significantly rescued Muv in *let-60 (gf); sgk-1 (lf)* worms ( **Fig. 1C** ) but had no effect on *let-60 (gf)* single mutant worms, *let-60 (gf); daf-2 (lf)* worms, or *let-60 (gf); daf-2 (lf); daf-16 (lf)* worms, indicating that an *sgk-1* specific pathway regulating Ras/MAPK signaling may involve *hpk-1 .* We thus predict that one function of *sgk-1* is to suppress *hpk-1* activity or expression, parallel to negative regulation of *daf-16* by *akt-1* and *2* . However, *hpk-1* is unlikely to explain the incomplete rescue of Muv in *let-60 (gf); daf-2 (lf); daf-16 (lf)* worms, as RNAi of *hpk-1* in this triple mutant background did not yield a further increase in Muv penetrance to *let-60 (gf)* single mutant levels; or, that its effect still requires active *daf-16* .

Factors which are inhibited by IGF signaling, like *daf-16* , should rescue Muv in the suppressed *let-60 (gf); daf-2 (lf)* or *let-60 (gf); sgk-1 (lf)* backgrounds when knocked down by RNAi. However, those that are positively regulated by IGF signaling would only show an effect in *let-60 (gf)* single mutant worms when depleted. We found several genes that phenocopied *daf-2 (lf)* and *sgk-1 (lf)* when knocked down in *let-60 (gf)* worms by RNAi. Among these were the *skn-1* /Nrf transcription factor and its target *gcs-1 /* GCLC (Tullet et al. 2008; An and Blackwell 2003), as well as the paraquat responsive transcription factor *pqm-1* which was previously shown to complement *daf-16* in regulation of longevity by *daf-2* and is regulated by *sgk-1* through phosphorylation (Tepper et al. 2013; Dowen et al. 2016). We found that RNAi knockdown or genetic ablation of *pqm-1* modestly suppressed Muv of *let-60 (gf)* worms ( **Fig. 1D and E** ). One enhancer of the *let-60 (gf)* Muv phenotype, the ubiquitously transcribed on X homolog *utx-1* had no effect on *let-60 (gf); daf-2 (lf)* or *let-60 (gf); sgk-1 (lf)* worms, indicating that its effect also depends on having functional IGF signaling.

While *pqm-1* has no obvious mammalian homolog, we reasoned that we may nonetheless identify conserved regulators of Ras signaling among its transcriptional targets, as a ChIP-seq dataset for *pqm-1* was available. We thus performed a second RNAi screen in *let-60 (gf)* as well as *let-60 (gf); pqm-1 (lf)* worms targeting genes which were bound in their promoter regions by PQM-1 *.* Among the top enhancers of Muv in both backgrounds, we identified *nhl-2* as previously reported (Hammell et al. 2009) as well as *ceh-18* which has known vulval development phenotypes in several other genetic backgrounds (Parry, Xu, and Ruvkun 2007). Notably, we identified an uncharacterized gene *F22D6.2* , with no known phenotypes, that enhanced Muv in *let-60 (gf)* worms but not in *pqm-1 (lf)* worms. Furthermore, *F22D6.2* was well-conserved in its AN1 domain encoding region with mammalian *ZFAND3, 5* and *6* genes (van Kempen et al. 2023). An existing allele, *ok1685 ,* which is a partial deletion of the 5' region encompassing the first exon (encoding the N-terminal A20 Zinc finger domain) and a small part of the second exon yielded no phenotype when crossed to *let-60 (gf)* worms ( **Fig. 1 G-I** ). We tagged both wild type *F22D6.2* as well as the *ok1685* allele with FLAG at the C-terminus and confirmed that a protein is translated from the *ok1685* allele ( **Fig. 1H** ), which likely explains the lack of phenotype observed. Indeed, generating a full coding sequence deletion of *F22D6.2* by CRISPR/Cas9 ( *on33* allele) phenocopied the *F22D6.2* RNAi enhancement of the Muv phenotype of *let-60 (gf)* worms ( **Fig. 1 I, J** ). Upon further work with *F22D6.2 ( on33 )* worms, we noted an unusual multigenerational germline feminization phenotype at higher temperatures, leading us to name this gene and its paralog *F56F3.4* as multigenerational sterility and temperature regulated, *mstr-1* and *mstr-2* , respectively. This, we studied in detail (Eroglu et al. 2024).

## Methods

*Caenorhabditis elegans* worms were maintained at 20 ^o^ C on nematode growth medium (NGM) plates, and fed OP50 *Escherichia coli* as previously described (Brenner 1974). HT115 *E. coli* clones carrying RNAi vectors for the indicated gene (Kamath et al. 2003) were grown overnight (16h at 37 ^o^ C) in liquid LB medium with ampicillin (100 μg/mL) and tetracycline (10 μg/mL) selection, then induced for 6h with IPTG (0.5 mM) before being concentrated 10-fold and seeded onto NGM plates supplemented with 0.25 mM IPTG and 25 μg/ml carbenicillin. Embryos obtained by bleach synchronization were plated on RNAi bacterial lawns (50-100 embryos per treatment), grown for 3-4 days at 20 ^o^ C and then scored as adults for penetrance of the Muv phenotype. Control refers to RNAi towards the non-expressed pseudogene *Y95B8A_84.g* (Klosin et al. 2017) *.* CRISPR/Cas9 was performed as described (Eroglu, Yu, and Derry 2023), by injection of pre-assembled Cas9-crRNA with single-stranded DNA repair templates. Vulval induction was scored as described (Subramanian et al. 2021), by quantifying the number of VPC daughter cells at L4, assigning a score of 0.5 for 3-4 daughters and 1.0 for 5-8 daughters. Western blot was performed by picking 20 adult worms directly into 1x sample buffer (62.5mM Tris pH 6.8, 2% SDS, 50mM DTT, 10% glycerol, with 0.002% bromophenol blue), incubating at 95 ^o^ C for 15 minutes, followed by electrophoretic separation on BioRad TGX pre-cast gels before being transferred to PVDF membranes. Blocking was for 30 mins with 5% nonfat milk powder in TBS with 0.1% tween-20, and detection was with 1:1000 mouse anti-FLAG (M2, Sigma F3165) primary antibody in blocking buffer and 1:5000 goat-anti- mouse IgG (H+L) HRP conjugated secondary antibody (ThermoFisher 31430) in blocking buffer.

## Reagents

**Table:** 

**Strain**	**Genotype**	**Available from**
N2	*Caenorhabditis elegans* WT	CGC
MT2124	*let-60 ( n1046 ) IV *	CGC
VC1240	*mstr-1 ( ok1685 ) I *	CGC
WD342	*let-60 ( n1046 ) IV; sgk-1 ( ok538 ) X *	WB Derry
WD315	*daf-2 ( e1370 ) III; let-60 ( n1046 ) IV *	WB Derry
WD347	*let-60 ( n1046 ) IV; daf-2 ( e1370 ) III; daf-16 ( mgDf47 ) I *	WB Derry
WD554	*let-60 ( n1046 ) IV; pqm-1 ( ok485 ) II *	WB Derry
WD598	*mstr-1 ( on33 ) I *	WB Derry
WD612	*let-60 ( n1046 ) IV; mstr-1 ( on33 ) I *	WB Derry

## References

[R1] An JH, Blackwell TK (2003). SKN-1 links C. elegans mesendodermal specification to a conserved oxidative stress response.. Genes Dev.

[R2] Battu G, Hoier EF, Hajnal A (2003). The C. elegans G-protein-coupled receptor SRA-13 inhibits RAS/MAPK signalling during olfaction and vulval development.. Development.

[R3] Beitel GJ, Tuck S, Greenwald I, Horvitz HR (1995). The Caenorhabditis elegans gene lin-1 encodes an ETS-domain protein and defines a branch of the vulval induction pathway.. Genes Dev.

[R4] Brenner S (1974). The genetics of Caenorhabditis elegans.. Genetics.

[R5] Dowen RH, Breen PC, Tullius T, Conery AL, Ruvkun G (2016). A microRNA program in the C. elegans hypodermis couples to intestinal mTORC2/PQM-1 signaling to modulate fat transport.. Genes Dev.

[R6] Eisenmann DM, Kim SK (1997). Mechanism of activation of the Caenorhabditis elegans ras homologue let-60 by a novel, temperature-sensitive, gain-of-function mutation.. Genetics.

[R7] Eroglu M, Yu B, Derry WB (2023). Efficient CRISPR/Cas9 mediated large insertions using long single-stranded oligonucleotide donors in C. elegans.. FEBS J.

[R8] Eroglu M, Zocher T, McAuley J, Webster R, Xiao MZX, Yu B, Mok C, Derry WB (2024). Noncanonical inheritance of phenotypic information by protein amyloids.. Nat Cell Biol.

[R9] Ferguson EL, Horvitz HR (1985). Identification and characterization of 22 genes that affect the vulval cell lineages of the nematode Caenorhabditis elegans.. Genetics.

[R10] Hall, Mathew Adam. 2014. “Insulin Signaling Promotes Ras/MAPK Cascade Activity by Inhibiting the FOXO Transcription Factor DAF-16.” MSc Thesis, Toronto: University of Toronto. http://hdl.handle.net/1807/82644.

[R11] Hammell CM, Lubin I, Boag PR, Blackwell TK, Ambros V (2009). nhl-2 Modulates microRNA activity in Caenorhabditis elegans.. Cell.

[R12] Hill RJ, Sternberg PW (1992). The gene lin-3 encodes an inductive signal for vulval development in C. elegans.. Nature.

[R13] Kamath RS, Fraser AG, Dong Y, Poulin G, Durbin R, Gotta M, Kanapin A, Le Bot N, Moreno S, Sohrmann M, Welchman DP, Zipperlen P, Ahringer J (2003). Systematic functional analysis of the Caenorhabditis elegans genome using RNAi.. Nature.

[R14] van Kempen M, Kim SS, Tumescheit C, Mirdita M, Lee J, Gilchrist CLM, Söding J, Steinegger M (2023). Fast and accurate protein structure search with Foldseek.. Nat Biotechnol.

[R15] Klosin A, Reis K, Hidalgo-Carcedo C, Casas E, Vavouri T, Lehner B (2017). Impaired DNA replication derepresses chromatin and generates a transgenerationally inherited epigenetic memory.. Sci Adv.

[R16] Muñoz-Maldonado C, Zimmer Y, Medová M (2019). A Comparative Analysis of Individual RAS Mutations in Cancer Biology.. Front Oncol.

[R17] Nakdimon I, Walser M, Fröhli E, Hajnal A (2012). PTEN negatively regulates MAPK signaling during Caenorhabditis elegans vulval development.. PLoS Genet.

[R18] Parry DH, Xu J, Ruvkun G (2007). A whole-genome RNAi Screen for C. elegans miRNA pathway genes.. Curr Biol.

[R19] Perrin AJ, Gunda M, Yu B, Yen K, Ito S, Forster S, Tissenbaum HA, Derry WB (2012). Noncanonical control of C. elegans germline apoptosis by the insulin/IGF-1 and Ras/MAPK signaling pathways.. Cell Death Differ.

[R20] Schindler AJ, Sherwood DR (2013). Morphogenesis of the caenorhabditis elegans vulva.. Wiley Interdiscip Rev Dev Biol.

[R21] Sternberg PW, Van Auken K, Wang Q, Wright A, Yook K, Zarowiecki M, Arnaboldi V, Becerra A, Brown S, Cain S, Chan J, Chen WJ, Cho J, Davis P, Diamantakis S, Dyer S, Grigoriadis D, Grove CA, Harris T, Howe K, Kishore R, Lee R, Longden I, Luypaert M, Müller HM, Nuin P, Quinton-Tulloch M, Raciti D, Schedl T, Schindelman G, Stein L (2024). WormBase 2024: status and transitioning to Alliance infrastructure.. Genetics.

[R22] Subramanian A, Hall M, Hou H, Mufteev M, Yu B, Yuki KE, Nishimura H, Sathaseevan A, Lant B, Zhai B, Ellis J, Wilson MD, Daugaard M, Derry WB (2021). Alternative polyadenylation is a determinant of oncogenic Ras function.. Sci Adv.

[R23] Tepper RG, Ashraf J, Kaletsky R, Kleemann G, Murphy CT, Bussemaker HJ (2013). PQM-1 complements DAF-16 as a key transcriptional regulator of DAF-2-mediated development and longevity.. Cell.

[R24] Yoder JH, Chong H, Guan KL, Han M (2003). Modulation of KSR activity in Caenorhabditis elegans by Zn ions, PAR-1 kinase and PP2A phosphatase.. EMBO J.

